# Supplementary material for: Accurate, scalable and integrative haplotype estimation
Source: Nat Commun. 2019 Nov 28;10:5436. doi: 10.1038/s41467-019-13225-y (PMC6882857; doi:10.1038/s41467-019-13225-y)
Supplement: Supplementary file 1 — Supplementary Information [file 41467_2019_13225_MOESM1_ESM.pdf]

Supplementary Information

Accurate scalable and integrative haplotype estimation

Delaneau et al.

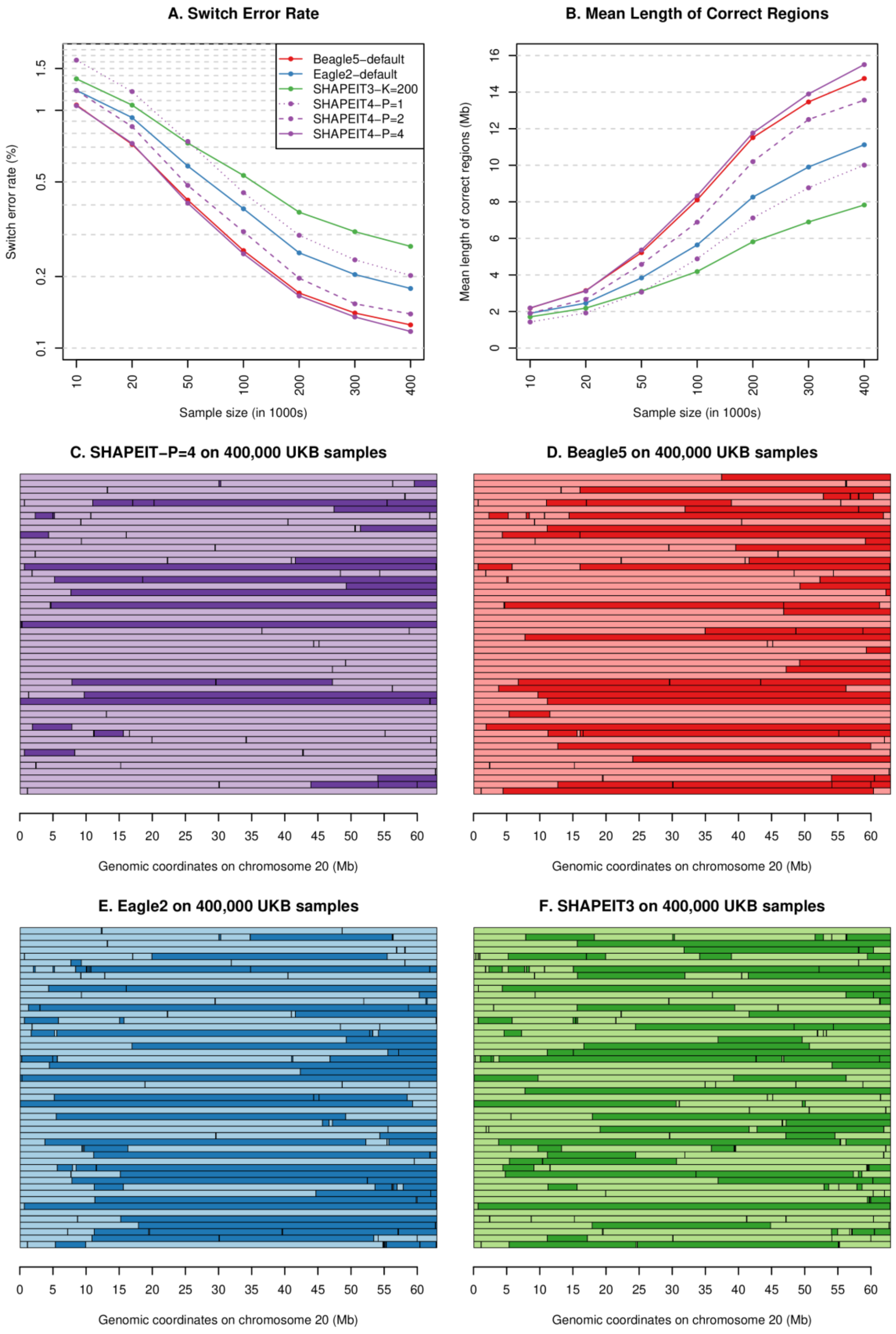

<u>Supplementary Figure 1:</u> Switch errors in UKB. Switch error rates (**A**) and average genomic distances between switch errors (i.e. size of the regions being correctly phased; **B**) as a function of sample size and tested method. Genomic locations of the switch errors on chromosome 20 across 50 UKB samples used for validation when inferred using SHAPEIT4-P=4 (**C**), Beagle5 (**D**), Eagle2 (**E**) an SHAPEIT3 (**F**) on 400,00UKB samples. Any switch between light and dark colour stands for a switch error.

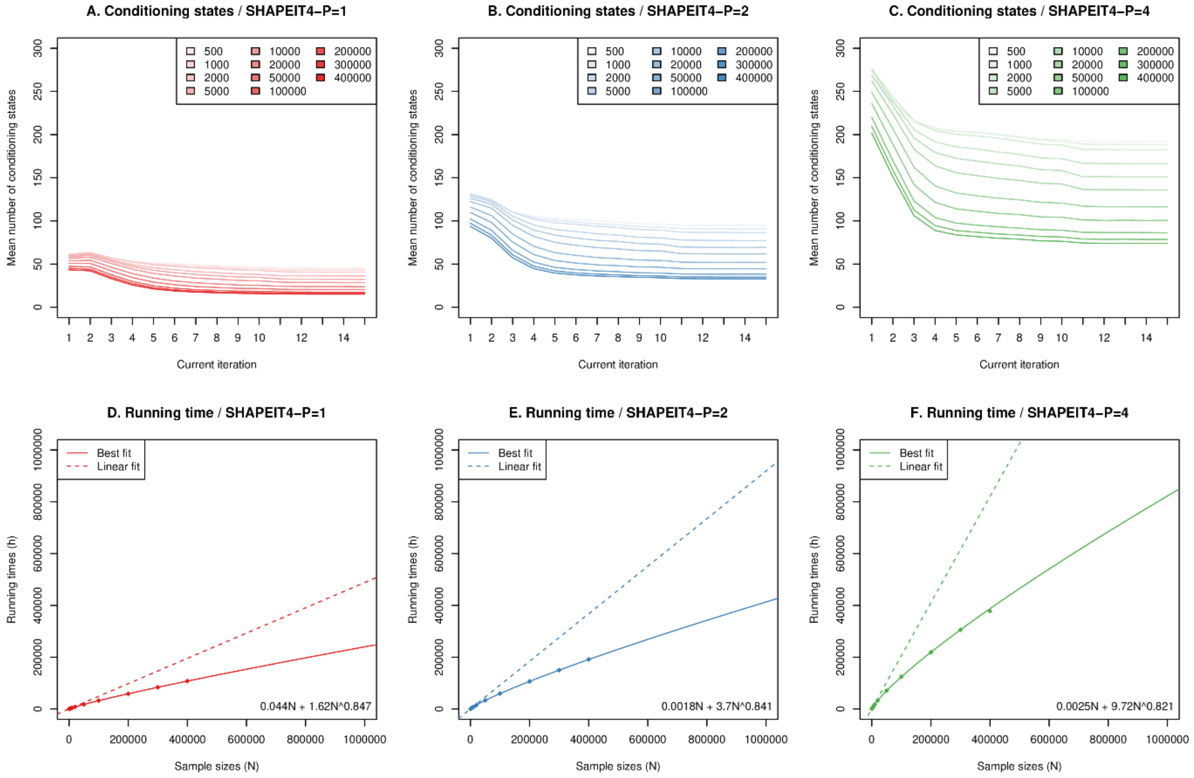

<u>**Supplementary Figure 2:**</u> **SHAPEIT4 running time and model size.** Variation of the mean numbers of conditioning haplotypes used by SHAPEIT4 as a function of the iteration (from 0 to 15) for multiple subsets of the UKB data set (from 500 to 400,000 individuals) and across multiple SHAPEIT4 parameter values (**A-C**). Running times as a function of UKB sample size for SHAPEIT4 when run with P=1 (**A**), P=2 (**B**) and P=4 (**C**). The dots show the measured running times. The plain lines show the fitted function (detailed on the bottom right corner) on the available data points. The dashed lines show the linear fit obtained when fitted to the first two data points.

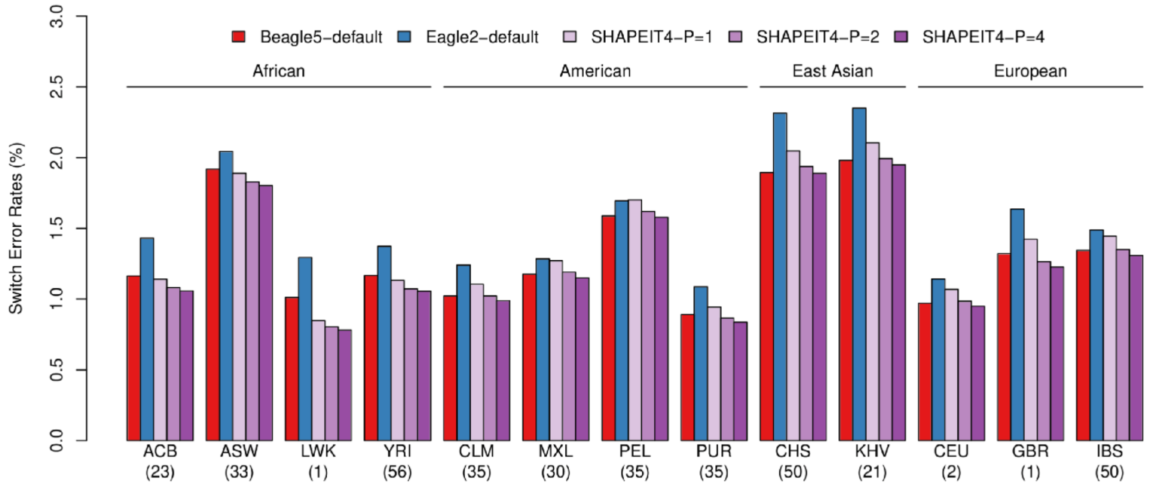

Super Pop.	Pop.	Pop. Description	#samples (Total)	#samples (Validation)
African	ACB	*African Caribbeans in Barbados*	56	23
	ASW	*Americans of African Ancestry in SW USA*	51	33
	LWK	*Luhya in Webuye, Kenya*	99	1
	MKK	*Maasai in Kinyawa, Kenya*	31	0
	YRI	*Yoruba in Ibadan, Nigeria*	62	56
American	CLM	*Colombians from Medellin, Colombia*	39	35
	MXL	*Mexican Ancestry from Los Angeles, USA*	40	30
	PEL	*Peruvians from Lima, Peru*	35	35
	PUR	*Puerto Ricans from Puerto Rico*	41	35
East Asian	CDX	*Chinese Dai in Xishuangbanna, China*	100	0
	CHB	*Han Chinese in Beijing, China*	101	0
	CHS	*Southern Han Chinese*	50	50
	JPT	*Japanese in Tokyo, Japan*	100	0
	KHV	*Kinh in Ho Chi Minh City, Vietnam*	80	21
European	CEU	*Utah Residents (CEPH)*	100	2
	FIN	*Finnish in Finland*	100	0
	GBR	*British in England and Scotland*	100	1
	IBS	*Iberian Population in Spain*	50	50
	TSI	*Toscani in Italia*	100	0
Other	GIH	*Gujarati Indian from Houston, USA*	100	0
		Total	1,435	372

Supplementary Figure 3: **Accuracy across multiple ancestries.** Switch error rates for all tested methods stratified across 13 different populations (top panel). Details of the populations used in this benchmark (bottom panel). Data on chromosome 20 was used here for a super set of 2,141 samples from 1,000 Genomes including multiple trios and duos. Only unrelated samples (n=1,063) and trio/duo children (n=372) were phased using the various methods in this benchmark (n=1,435) and the trio/duo parents (n=706) were used to evaluate the switch error rates in the phased trio/duo children.

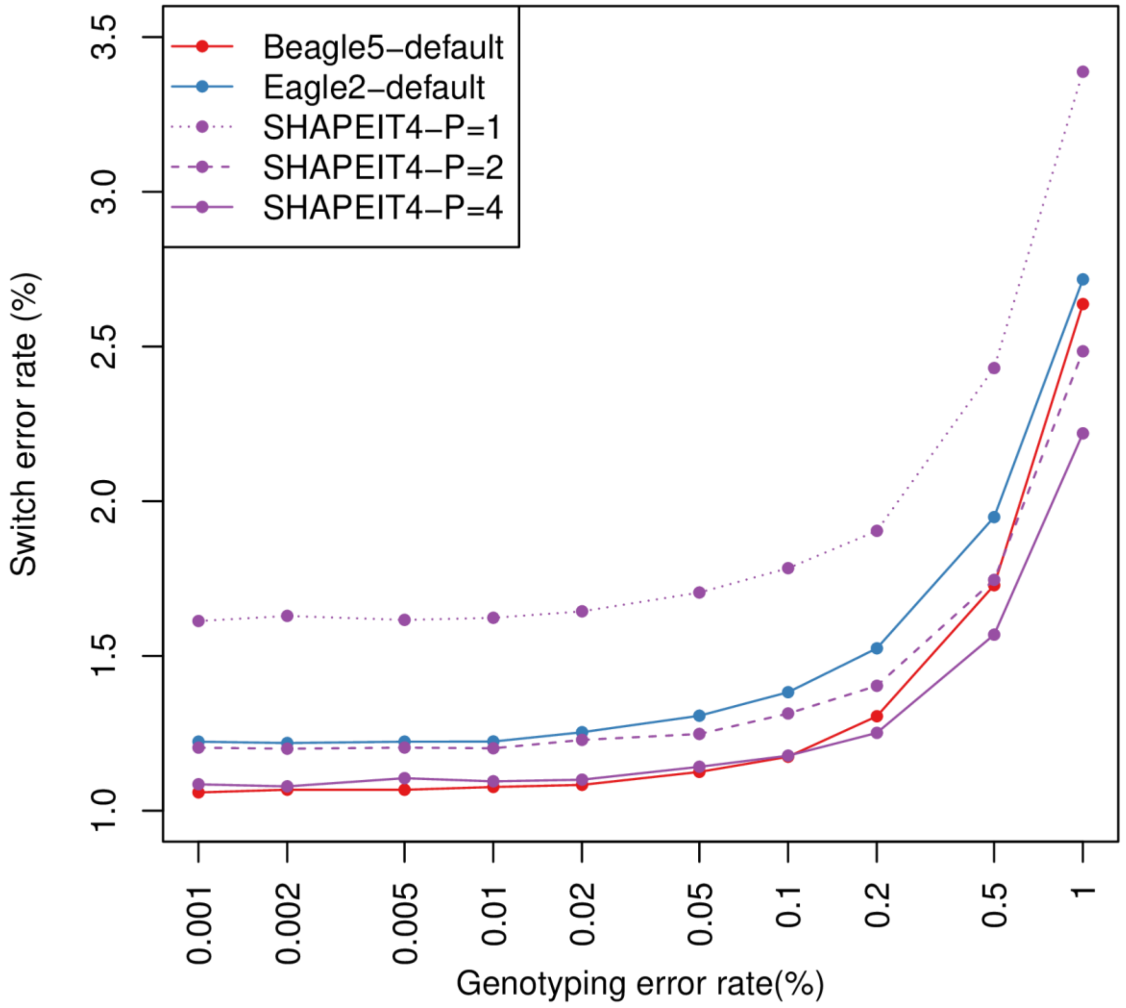

Supplementary Figure 4: Accuracy versus genotyping error rates. Switch error rates for all tested methods is shown as a function of the genotyping error rate. The dataset used here comprises 10,000 UKB samples in which increasing percentages of genotype errors were introduced. Switch error rates for the various methods were computed on 500 trio children, only between genotypes at which no errors were introduced.

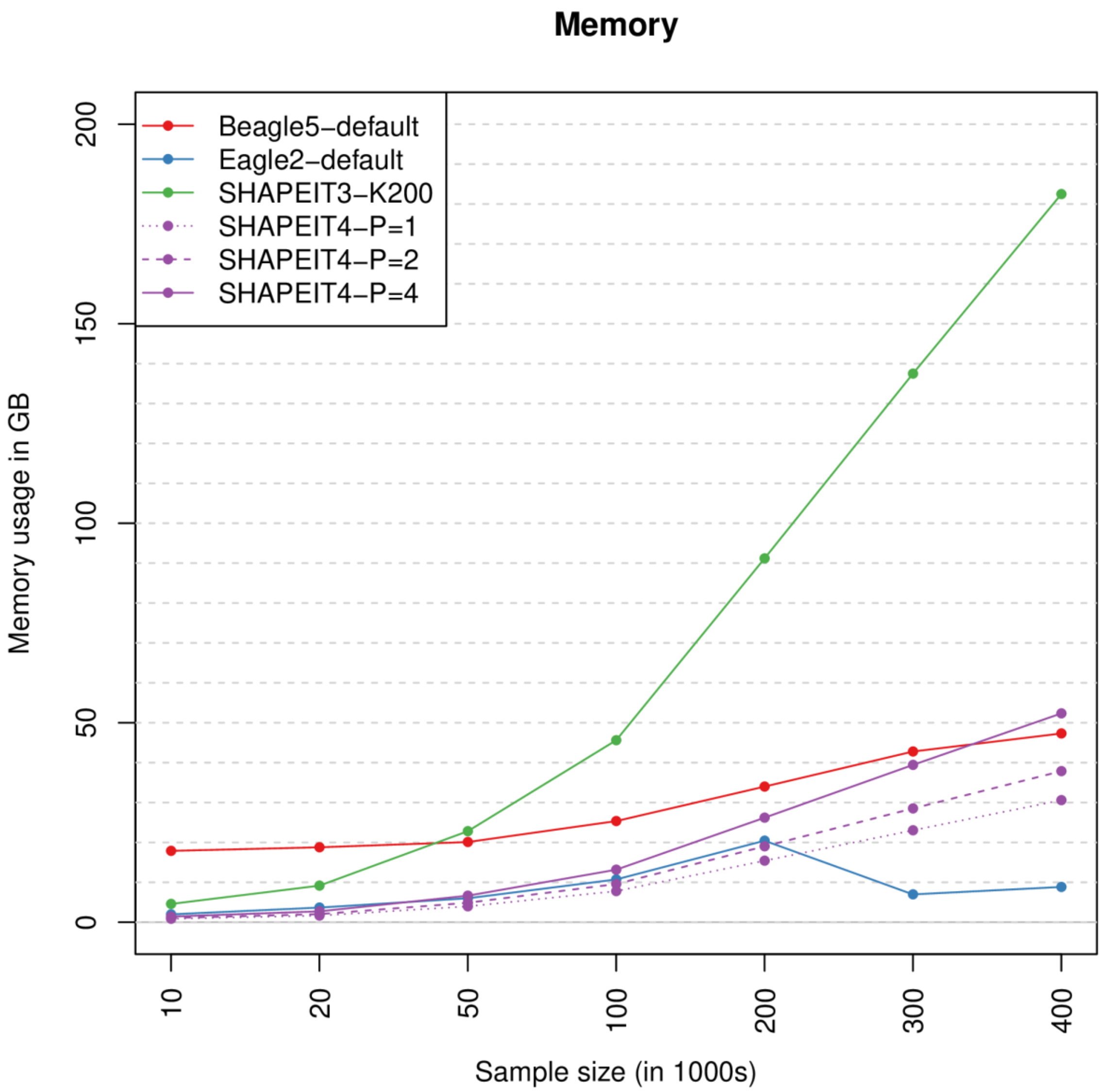

<u>**Supplementary Figure 5:**</u> **Memory usage.** Memory usage in GigaBytes measured by the Linux time command as a function of the sample size and the method tested.

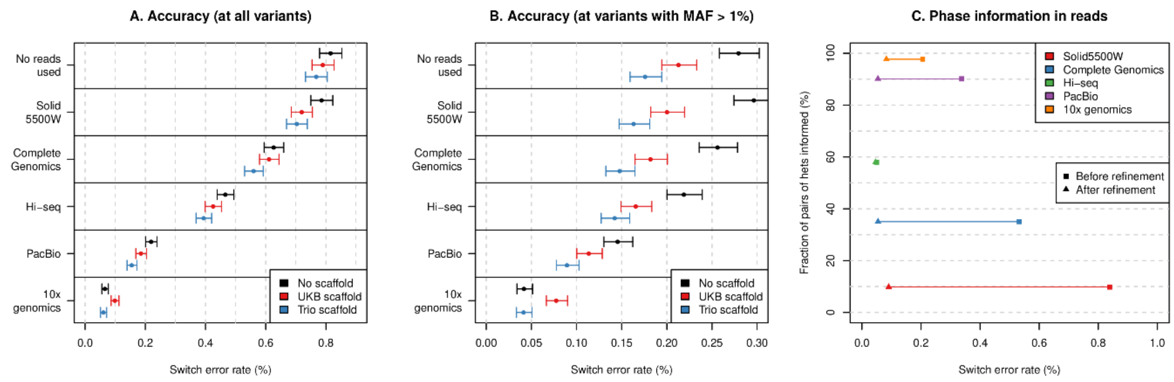

<u>Supplementary Figure 6:</u> Phasing performance on high coverage sequence data (GIAB). (**A**) Switch error rates with 95% binomial confidence intervals for each combination tested of sequencing reads and haplotype scaffold. (**B**) Same information than in (A) measured only at variants with minor allele frequency (MAF) above 1%. (**C**) Switch error rates measured only at variants belonging to phase sets (i.e. haplotype assembly) before (squares) and after (triangles) refinement by SHAPEIT4. Results are shown here assuming 1% error rate in the phase sets.

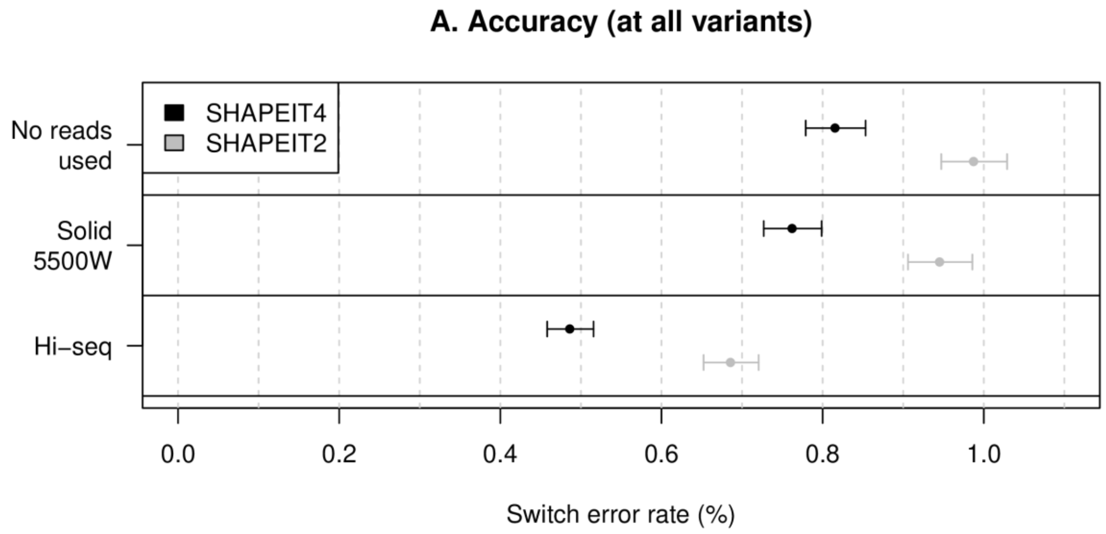

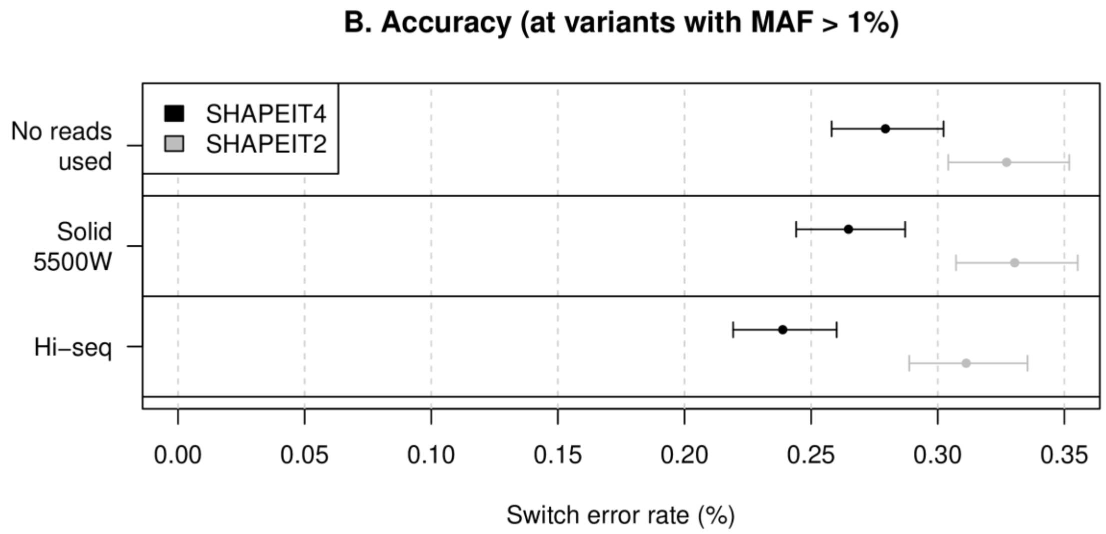

Supplementary Figure 7: Comparison of SHAPEIT4 and SHAPEIT2 on high coverage sequence data (GIAB). (**A**) Switch error rates with 95% binomial confidence intervals for multiple types of sequencing reads. (**B**) Same information than in (A) measured only at variants with minor allele frequency (MAF) above 1%. Results are only shown for sequencing data on which we could run the SHAPEIT2 model, the other ones present base qualities encoded (if present at all) in a format that the SHAPEIT2 model cannot directly handle.

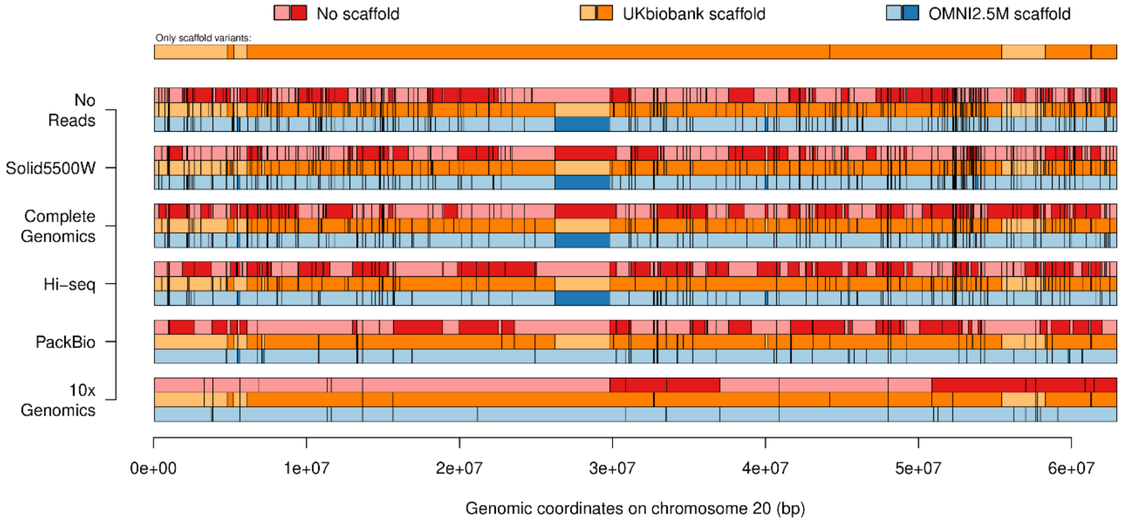

<u>Supplementary Figure 8:</u> Switch errors in GIAB. Genomic locations of the switch errors on chromosome 20 for each one of the 18 tested configurations. Switches between dark and light colour represent switch errors. The top row shows the switch errors only between variants in the overlap with UKB when NA12878 is phased against UKB (i.e. accuracy of the UKB scaffold).

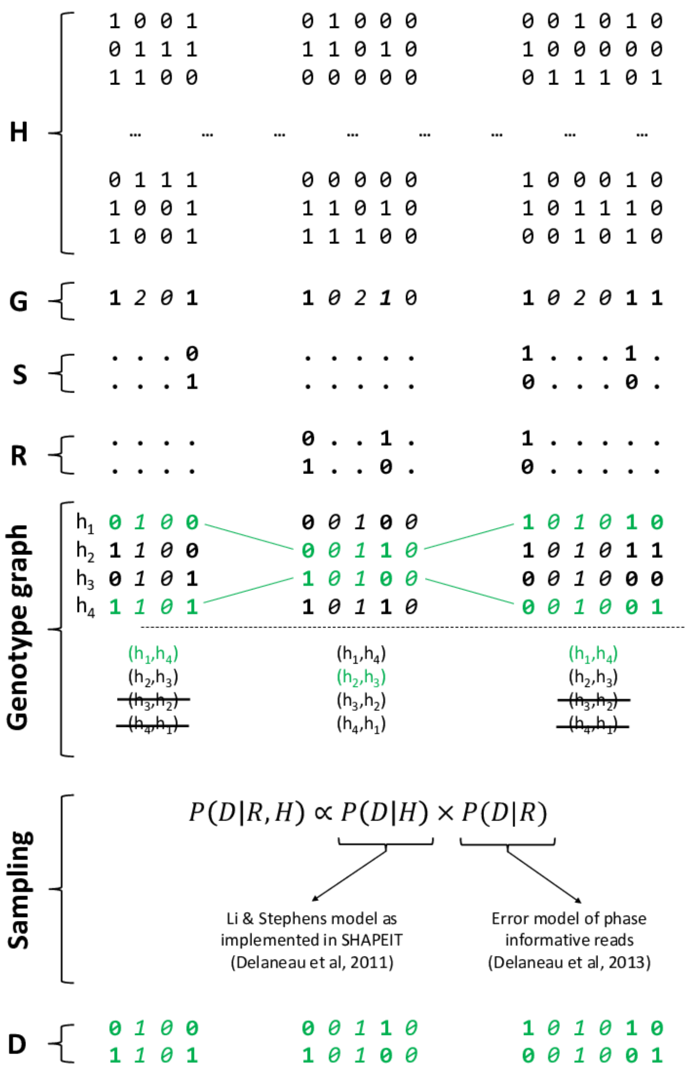

<u>Supplementary Figure 9:</u> Overview of the SHAPEIT4 genotype graph structure. From top to bottom. (1) The set **H** of conditioning haplotypes that were estimated for other samples in the dataset. This conditioning set is usually small as we pre-select informative haplotypes using PBWT. (2) The genotype data **G** available for an individual with 0, 1 and 2 corresponding to homozygous reference, heterozygous and homozygous alternative genotypes. Non heterozygous genotypes are italicized. (3) The haplotype scaffold **S** available for this individual with three pre-phased heterozygous genotypes. (4) The phase set **R** available for this individual at three heterozygous genotypes. (5) The genotype graph structure for **G** when assuming only two *unphased* heterozygous genotypes per segment (by default, it is three). Note that in the last segment, there are three heterozygous genotypes as two of them belong to the scaffold and do not require phase relative to each other. On the top panel, the four possible haplotypes per segment. On the bottom panel, the pairs of haplotypes consistent with both **G** and **S**. Pairs that are not consistent with **S** are crossed out. (6) Distribution from which haplotypes pairs are sampled through the genotype graph. The transition probabilities, P(D|H), are computed using the Li and Stephens model as described in the SHAPEIT1 paper (Delaneau et al, Nature Methods 2011). The transition probabilities, P(D|R), are computed using a simple error model as described in the method section: transitions between segments that are consistent with the reads are given a high probability (e.g. 0.999) while those that are not a low probability (e.g. 0.001). Details on the sampling from P(D|H, R) can be found in Delaneau et al, AJHG 2013. (7) An example of sampled haplotype pairs D for G is shown in green.
