# Supplementary material for: Accurate, scalable and integrative haplotype estimation
Source: Nat Commun. 2019 Nov 28;10:5436. doi: 10.1038/s41467-019-13225-y (PMC6882857; doi:10.1038/s41467-019-13225-y)
Supplement: Supplementary file 3 — Reporting Summary [file 41467_2019_13225_MOESM3_ESM.pdf]

nature research

Corresponding author(s): Olivier Delaneau

Last updated by author(s): Sep 13, 2019

Reporting Summary

Nature Research wishes to improve the reproducibility of the work that we publish. This form provides structure for consistency and transparency in reporting. For further information on Nature Research policies, see Authors & Referees and the Editorial Policy Checklist.

Statistics

For all statistical analyses, confirm that the following items are present in the figure legend, table legend, main text, or Methods section.

n/a	Confirmed	
☐	☒	The exact sample size (*n*) for each experimental group/condition, given as a discrete number and unit of measurement
☒	☐	A statement on whether measurements were taken from distinct samples or whether the same sample was measured repeatedly
☒	☐	The statistical test(s) used AND whether they are one- or two-sided<br>*Only common tests should be described solely by name; describe more complex techniques in the Methods section.*
☒	☐	A description of all covariates tested
☒	☐	A description of any assumptions or corrections, such as tests of normality and adjustment for multiple comparisons
☐	☒	A full description of the statistical parameters including central tendency (e.g. means) or other basic estimates (e.g. regression coefficient) AND variation (e.g. standard deviation) or associated estimates of uncertainty (e.g. confidence intervals)
☒	☐	For null hypothesis testing, the test statistic (e.g. *F*, *t*, *r*) with confidence intervals, effect sizes, degrees of freedom and *P* value noted<br>*Give P values as exact values whenever suitable.*
☐	☒	For Bayesian analysis, information on the choice of priors and Markov chain Monte Carlo settings
☐	☒	For hierarchical and complex designs, identification of the appropriate level for tests and full reporting of outcomes
☒	☐	Estimates of effect sizes (e.g. Cohen's *d*, Pearson's *r*), indicating how they were calculated

Our web collection on statistics for biologists contains articles on many of the points above.

Software and code

Policy information about availability of computer code

Data collection	No software was used.
Data analysis	Data management:<br>bcftools v1.4 (http://www.htslib.org)<br><br>Phasing:<br>SHAPEIT v4.0 (https://odelaneau.github.io/shapeit4/)<br>Beagle5.0 (beagle.14May18.ff7.jar; https://faculty.washington.edu/browning/beagle/beagle.html)<br>Eagle v2.4 (https://data.broadinstitute.org/alkesgroup/Eagle/)<br>SHAPEIT v3 (https://jmarchini.org/shapeit3/)

For manuscripts utilizing custom algorithms or software that are central to the research but not yet described in published literature, software must be made available to editors/reviewers. We strongly encourage code deposition in a community repository (e.g. GitHub). See the Nature Research guidelines for submitting code & software for further information.

Data

Policy information about availability of data

All manuscripts must include a data availability statement. This statement should provide the following information, where applicable:

- Accession codes, unique identifiers, or web links for publicly available datasets
- A list of figures that have associated raw data
- A description of any restrictions on data availability

We used the full release of the UK biobank (http://www.ukbiobank.ac.uk/). This work was conducted under UK Biobank project 35520. We used the following release of the GIAB (ftp://ftp-trace.ncbi.nlm.nih.gov/giab/) and this release of the phase 3 of the 1000 Genomes project (ftp://ftp.1000genomes.ebi.ac.uk). We downloaded this version of the larger set of 1000 Genomes samples genotyped on Illumina OMNI2.5M (ftp://ftp.1000genomes.ebi.ac.uk). The sequencing data we

used for GIAB are available from this location (ftp://ftp-trace.ncbi.nlm.nih.gov/giab/ftp/data/NA12878/) and 10X Genomics VCF populated with PS field (BAM). ftp://ftp-trace.ncbi.nlm.nih.gov/giab/ftp/data/NA12878/10Xgenomics_ChromiumGenome_LongRanger2.1_09302016/NA12878_hg19/NA12878_hg19_phased_variants.vcf.gz). All other relevant data is available upon request.

Field-specific reporting

Please select the one below that is the best fit for your research. If you are not sure, read the appropriate sections before making your selection.

☒ Life sciences ☐ Behavioural & social sciences ☐ Ecological, evolutionary & environmental sciences

For a reference copy of the document with all sections, see nature.com/documents/nr-reporting-summary-flat.pdf

Life sciences study design

All studies must disclose on these points even when the disclosure is negative.

Sample size	No sample-size calculation were performed. We used for each dataset, the maximum number of samples available.
Data exclusions	We remove some genetic variants in the datasets we used according to standard quality control metrics.
Replication	We replicated our measures when possible across two datasets (UK Biobank and 1000 Genomes).
Randomization	As we do not compare two groups of samples, no randomization was needed.
Blinding	As we do not compare two groups of samples, no blind group assinment was needed.

Reporting for specific materials, systems and methods

We require information from authors about some types of materials, experimental systems and methods used in many studies. Here, indicate whether each material, system or method listed is relevant to your study. If you are not sure if a list item applies to your research, read the appropriate section before selecting a response.

Materials & experimental systems

n/a	Involved in the study	
☒	☐	Antibodies
☒	☐	Eukaryotic cell lines
☒	☐	Palaeontology
☒	☐	Animals and other organisms
☒	☐	Human research participants
☒	☐	Clinical data

Methods

n/a	Involved in the study	
☒	☐	ChIP-seq
☒	☐	Flow cytometry
☒	☐	MRI-based neuroimaging
